# Age Related Differences in Body Mass Index Among Young Amazonian Peruvian Children

**DOI:** 10.51894/001c.164355

**Published:** 2026-07-31

**Authors:** Alyssa Murphy, Julia Hasan, Bhavya Thotakura, Rene Hinojosa, Jodi Flanders

**Affiliations:** 1 College of Osteopathic Medicine, Michigan State University

## 31

### INTRODUCTION

The World Health Organization (WHO) developed growth charts to assess child growth and development worldwide. Pediatric growth charts often emphasize weight and height measurements. These measurements alone do not provide sufficient insight into conditions such as obesity or wasting. Body mass index (BMI), combining weight and height, can be more appropriate indicators of nutritional status. The WHO BMI growth charts provide an international reference used to assess childhood undernutrition and obesity risk. Recorded trends can be useful in estimating long-term health implications.

### OBJECTIVES

The aim is to compare the BMI values of Peruvian children aged 3 months to 5 years living in the Amazon region. BMI values will be analyzed by age group, comparing children younger than 2 years with those older than 2 years of age. It is hypothesized that there is a difference in body mass outcomes between these groups, with younger children being worse off in terms of BMI.

### METHODS

A cross-sectional sample of 150 cases was collected from children aged 0-5 years in Iquitos, Peru. Anthropometric measurements included weight, height, head circumference, mid-upper arm circumference, and triceps skinfold thickness measured using a calibrated caliper. Body mass index was calculated along BMI-for-age z-scores. To evaluate the agreement between measurements and WHO expected values, Bland-Altman plots were analyzed. Statistical comparison of BMIs between age groups was performed with t-tests.

### RESULTS

According to z-BMI values and WHO cutoffs, only 78.9% of children were classified as normal weight, 9.1% showed moderate wasting and 2.8% were severely wasted. Additionally, 6.4% were categorized as at risk of overweight and 2.8% as overweight, with no children meeting criteria for obesity. Bland–Altman analysis of differences between expected and measured values indicate that children are below expectations for weight and height (0.70 Kg and 0.39 cm). T-test analysis demonstrated both age groups were below expected BMI values, with younger children significantly lower than older children (*p* = 0.005).

### CONCLUSIONS

Children 0-5 years old are under the expected WHO body mass values, with weight and height less than expected. The presence of bias suggests that WHO standards may not be appropriate for this population.

